# Editorial: Oral health, aging, and inequalities: functional outcomes, nutrition, and quality of life in older adults

**DOI:** 10.3389/fdmed.2026.2034563

**Published:** 2026-10-29

**Authors:** Roosevelt Silva Bastos, Takaaki Tomofuji, Naofumi Tamaki

**Affiliations:** 1 Department of Pediatric Dentistry, Orthodontics and Public Health, Faculty of Dentistry of Bauru, University of São Paulo, Bauru, Brazil; 2 Asahi University, Mizuho, Japan; 3 Department of Preventive Dentistry, Kagoshima University Graduate School of Medical and Dental Sciences, Kagoshima, Japan

**Keywords:** frailty, health inequalities, healthy aging, nutritional status, older adults, oral function, oral health, oral health-related quality of life

Population aging calls for approaches to oral health that address functional ability, nutritional well-being, and quality of life alongside the prevention and treatment of disease. Preserving natural teeth, maintaining comfortable and effective oral function, and supporting satisfactory prosthetic rehabilitation are relevant to how people eat, communicate, and participate in daily life. These dimensions must also be understood within the social conditions of aging. Differences in income, education, food security, and access to care can shape opportunities to maintain oral health throughout life and the resources available to manage oral problems in later years. An integrated perspective connecting dentistry, gerontology, nutrition, and public health is therefore essential to understanding oral health in aging populations.

The Research Topic “Oral Health, Aging, and Inequalities: Functional Outcomes, Nutrition, and Quality of Life in Older Adults” brings together 12 contributions examining complementary aspects of these relationships. Across community, institutional, and clinical settings, the collection explores oral conditions and frailty, nutritional vulnerability, adaptation to prosthetic rehabilitation, patient-reported outcomes, oral health literacy, and social participation. Contributions involving younger and middle-aged adults extend this discussion through a life-course perspective. Together, these articles connect clinical findings, functional outcomes, and social circumstances, while identifying questions that remain unresolved. This editorial synthesizes their contributions and outlines priorities for research and care that support oral function, well-being, and equity in aging populations.

## Oral health, nutrition, and frailty

Four contributions examine the relationships between oral health, nutritional vulnerability, and broader dimensions of health in later life. Lowenstein et al. propose a clinical framework linking polypharmacy, xerostomia, and nutrition, drawing attention to the potential consequences of medication-related oral dryness for eating and nutritional well-being. This framework provides a useful perspective alongside the population-based analysis by Pereira et al. Among community-dwelling older adults in Brazil, greater xerostomia symptom severity was associated with higher frailty scores, but the survey-weighted structural equation model did not support an indirect association through nutritional vulnerability. Considered together, these contributions highlight the clinical relevance of oral dryness while leaving open questions about the pathways connecting it with broader health outcomes.

Da et al. and Ulema extend this discussion to patient-reported and psychosocial dimensions. Among older adults in Shanghai, Da et al. found results consistent with a potential indirect association between the number of remaining teeth and frailty through oral health-related quality of life. In rural older adults in Türkiye, Ulema identified associations of oral frailty, food insecurity, and malnutrition with depressive symptoms, with exploratory analyses suggesting that malnutrition may contribute to the observed relationships. These proposed pathways remain hypotheses requiring longitudinal investigation, but they broaden the discussion by examining how oral conditions intersect with perceived impacts, access to food, and mental health.

These articles support attention to oral symptoms, dentition, nutritional status, and psychosocial circumstances within comprehensive assessment of older adults. The findings also suggest that nutritional vulnerability may not account for all observed associations between oral health and frailty. Their practical contribution is to encourage coordinated assessment across professional disciplines, while recognizing that older adults differ in their resources to obtain appropriate food, manage oral symptoms, and access continuing care.

## Rehabilitation, adaptation, and patient experience

Three contributions examine complementary dimensions of prosthetic rehabilitation, emphasizing functional adaptation, variation in outcomes over time, and patients’ experiences. In their systematic review, Shetty Ganesh et al. report preliminary findings suggesting that neuromuscular re-education, oral motor exercises, and biofeedback may support adaptation and masticatory performance in complete denture wearers. Zhang et al. add a longitudinal perspective by identifying three oral frailty trajectories during the year following implant-supported prosthesis delivery: rapid improvement followed by stability, gradual improvement followed by a plateau, and persistently elevated scores. Older age, a greater medication burden, and more missing teeth were associated with the persistent high-risk trajectory. These findings highlight variation in patients’ needs after rehabilitation and the potential value of continued assessment and individualized support.

Ceraulo et al. complement these functional perspectives by examining the experiences of complete denture wearers across functional, psychological, and social dimensions. Their findings draw attention to social difficulties that may receive insufficient attention when rehabilitation is evaluated primarily through technical or clinical criteria. Together, these contributions suggest that assessment of rehabilitation should encompass comfort, oral function, adaptation, and participation in daily life. They also identify questions for future research about which supportive strategies improve outcomes and how affordability, access to follow-up, and patients’ social circumstances influence the experience of rehabilitation.

## Social contexts and a life-course perspective

Three contributions situate oral health within the social resources and circumstances of later life. Song et al. identified distinct active ageing profiles among rural older adults in China, with poorer oral health-related quality of life in profiles characterized by lower overall active ageing, limited social integration, or reduced financial security. In nursing homes, Zhao et al. found heterogeneous oral health literacy profiles associated with oral frailty, drawing attention to differences in understanding, communication, and economic resources. Among older people in an underpopulated area of Japan, Zhu et al. observed an association between the number of remaining teeth and higher-level functional capacity that was attenuated after adjustment for social participation. Although this attenuation does not establish mediation, it invites further investigation of how oral health and social participation relate to the capacity for independent living. These studies emphasize the relevance of accessible communication, opportunities for participation, and services responsive to social disadvantage.

Two further contributions extend the collection beyond older populations. Asadollahi et al. examined oral health profiles and quality of life among men aged 40 years and older with andropause symptoms, identifying cross-sectional associations involving oral conditions, education, and socioeconomic context. Talwar et al. documented differences in caries experience across adults aged 21–50 years, with higher cumulative disease experience in older age groups. These studies broaden the discussion to oral health before old age and encourage investigation of how earlier disease experience and unequal opportunities for prevention and care relate to later outcomes. A life-course perspective also places responsibility for healthy oral aging within health services and social policy, alongside support for individual health practices.

## Limitations of the evidence and future directions

Several limitations define the conclusions that can be drawn from the collection. Most empirical contributions are cross-sectional, leaving temporal ordering, reverse causation, and residual confounding unresolved. Structural equation models and mediation analyses explore relationships consistent with hypothesized pathways but cannot establish causal mechanisms from these data. Cross-sectional age comparisons likewise cannot distinguish individual aging trajectories from cohort differences. Differences in sampling, study settings, and measurement constrain comparisons and generalization: general frailty, oral frailty, nutritional vulnerability, and quality of life represent related but distinct constructs. Latent profiles and trajectories require external validation before informing clinical classification. The longitudinal rehabilitation study provides information on change over time, but the absence of a comparison group limits conclusions about treatment effects. The systematic review is constrained by a small evidence base and concerns about risk of bias, while the complete denture patient-reported study used a small sample and an adapted questionnaire without formal validation.

Future research should prioritize longitudinal studies with repeated clinical, functional, nutritional, and patient-reported assessments, alongside rigorous evaluations of interprofessional care. Trials and implementation studies could examine whether coordinated oral care, medication review, nutritional support, and rehabilitation follow-up improve outcomes meaningful to older adults. Validated measures and clearly defined constructs would strengthen comparability across populations and settings. Equity should also become an explicit outcome of this research. The collection offers insights into social vulnerability, but direct evaluation of whether services reduce differences in access, function, and well-being remains a priority. Including older adults in intervention design and outcome selection would help ensure that proposed approaches address their priorities and remain feasible within everyday care.

The contributions to this Research Topic place oral health within a broader understanding of aging that encompasses function, nutrition, psychological well-being, and social participation. They support a research and care agenda attentive to individual needs and to the unequal resources available for maintaining health throughout life. Advancing this agenda will require collaboration across dentistry, gerontology, nutrition, and public health, with older adults contributing to the definition of meaningful outcomes and priorities for care. We thank the authors, reviewers, and editorial team for their contributions to this collection and hope that it encourages further research and dialogue on equitable approaches to oral health and healthy aging.

